# Identification of basement membrane-related biomarkers associated with the diagnosis of osteoarthritis based on machine learning

**DOI:** 10.1186/s12920-023-01601-z

**Published:** 2023-08-23

**Authors:** Xiaojing Huang, Hongming Meng, Zeyu Shou, Jiahuan Yu, Kai Hu, Liangyan Chen, Han Zhou, Zhibiao Bai, Chun Chen

**Affiliations:** 1 Department of Orthopedics, The First Affiliated Hospital of Wenzhou Medical University, Wenzhou City, 325000 Zhejiang Province China; 2 Wenzhou Medical University, Wenzhou City, 325000 Zhejiang Province China; 3 Key Laboratory of Intelligent Treatment and Life Support for Critical Diseases of Zhejiang Province, Wenzhou, 325000 Zhejiang China; 4 Zhejiang Engineering Research Center for Hospital Emergency and Process Digitization, Wenzhou, 325000 Zhejiang China

**Keywords:** Osteoarthritis, Immune, Machine learning, Basement membranes, Biological marker

## Abstract

**Background:**

Osteoarthritis is a very common clinical disease in middle-aged and elderly individuals, and with the advent of ageing, the incidence of this disease is gradually increasing. There are few studies on the role of basement membrane (BM)-related genes in OA.

**Method:**

We used bioinformatics and machine learning methods to identify important genes related to BMs in OA patients and performed immune infiltration analysis, lncRNA‒miRNA-mRNA network prediction, ROC analysis, and qRT‒PCR.

**Result:**

Based on the results of machine learning, we determined that LAMA2 and NID2 were the key diagnostic genes of OA, which were confirmed by ROC and qRT‒PCR analyses. Immune analysis showed that LAMA2 and NID2 were closely related to resting memory CD4 T cells, mast cells and plasma cells. Two lncRNAs, XIST and TTTY15, were simultaneously identified, and lncRNA‒miRNA‒mRNA network prediction was performed.

**Conclusion:**

LAMA2 and NID2 are important potential targets for the diagnosis and treatment of OA.

## Introduction

Osteoarthritis (OA) is a very common clinical disease in middle-aged and elderly individuals, and with the advent of ageing, the incidence of this disease is gradually increasing. OA has been reported to affect more than 500 million people worldwide (approximately 7% of the global population), especially the elderly (> 65 years of age). Due to the increase in the number of obese individuals, post-traumatic OA cases and early diagnosis, the incidence of OA in people under 65 years of age has been increasing [1]. The gradual loss of articular cartilage is the main feature in OA. The formation of osteophytes at joint margins and bone remodeling associated with subchondral bone sclerosis and bone marrow lesions are other symptoms of OA [2]. As the relevant mechanism of OA is not known at present, there is no effective way to stop or reverse the progression of OA to date [3, 4]. Therefore, it is important to explore diagnostic biomarkers related to OA for the diagnosis and treatment of OA.

Recently, with the development of high-flux genetic microarray analysis technology, we have been able to study the development of related diseases at the genetic level [5, 6]. At present, many markers related to OA have been found. For example, mitochondrial double-stranded RNA has been reported to regulate the stress response of chondrocytes to promote the development of osteoarthritis [7]. Recent studies by Shang et al. found that circHIPK3 is downregulated in osteoarthritic chondrocytes and cartilage. A potential treatment strategy for OA might target the circHIPK3/miR-30a-3p/PON2 axis, which regulates chondrocyte apoptosis via mitochondrial pathways [8]. Moreover, a study by Xinyue Hu et al. found that patients with osteoarthritis expressed significantly lower levels of TLR7, TCA1, MMP9, CXCL13, CXCL10, HLA-DRA and ADIPOQSPP1 [9]. However, the role of many genes in the diagnosis and treatment of OA remains unknown.

BMs are the oldest animal extracellular matrix, forming sheet-like structures, which are associated with a variety of diseases, such as cancer. A variety of functions are performed by these structures, including blood filtration, muscle homeostasis, storing growth factors and cytokines, controlling angiogenesis and tumor growth, maintaining skin integrity and neuromuscular structure, and maintaining skin integrity and neuromuscular structure [10]. Jayadev et al. [11] showed that more than 100 BM genes found in the Human Phenotype Ontology (HPO), Online Mendelian Inheritance in Man (OMIM) and Genomics England PanelApp databases were associated with disease phenotypes in the skeletal system (bone and joint), and several BM genes found in the OMIM database were associated with osteoarthritis. However, there are still few reports on the correlation of BMs-related genes in the development of OA. Therefore, we aimed to investigate the relationship between BM genes and OA to deepen our understanding and guide our diagnosis and treatment of OA. Therefore, in this paper, we first conducted the correlation analysis by bioinformatics methods. Then, machine learning was used to analyse their diagnostic value in OA. Finally, quantitative real-time PCR (qRT‒PCR) was used to verify the correlation. We provide new targets related to the development of OA.

## Materials and methods

### Identification of OA-Related BM-Differentially expressed genes (DEGs)

We used dataset GSE55235 (platform: GPL96, Affymetrix Human Genome U133A Array) in the GEO database as the experimental set, which included 10 joint tissue samples from healthy humans and 10 tissue samples from patients with OA.

We adopted R (version 3.6.3) for statistical analysis and visualization, downloaded the datasets from the GEO database through the GEO query package (version 2.54.1) [12], and removed the probes corresponding to multiple molecules. When a probe corresponding to the same molecule was encountered, only the probe with the largest signal value was retained. The normalize Between Arrays function of the limma package (version 3.42.2) was then used to normalize the data again. Sample normalization was assessed by a box plot. The PCA chart and the UMAP chart were used to view the clustering between the sample groups, and then, the difference analysis between the two groups was performed using the limma package.

In this study, we chose a p value less than 0.05 and a |log FC| value greater than 0.5 for the standard figure generating the volcano. The visualization package ggplot2 (version 3.3.3) was used. The Complex Heatmap package (version 2.2.0) was used for heatmap visualization of the top 20 genes with high and low expression [13]. Basement membrane-related genes were obtained from previous studies [11], and BM-related DEGs were obtained by Venn diagram intersection.

### Gene ontology (GO) and kyoto encyclopedia of genes and genomes (KEGG) pathway enrichment analysis

We used the org.Hs.eg.db package (version 3.10.0) for ID conversion and the clusterProfiler package (version 3.14.3) for enrichment analysis [14]. It can provide biological process (BP), cellular component (CC), molecular function (MF) and pathway enrichment information (from KEGG database [15]). We then used the clusterProfiler package (version 3.14.3) for analysis of the selected data and the ggplot2 package (version 3.3.3) for visualization.

### Hub gene determination

The screened BM-related DEGs were used to construct a PPI network using the online analysis website STRING (https://string-db.org/) [16], the minimum required interaction score was set as 0.400, and the PPI data were derived. Then, the MCC algorithm of the cytoHubba plugin in Cytoscape (version 3.9.1) was used to screen hub genes, and the top 20 genes were obtained.

### Identification of candidate diagnostic markers

We used two kinds of machine learning to predict and intersect the prediction results with the HUB gene to determine the final diagnostic markers. In the LASSO analysis, forecast accuracy is improved by regularizing a regressive analytical arithmetic. With the glmnet package in R, LASSO regressive arithmetic was utilized to find the genes related to OA and healthy specimens’ discriminative power. Support vector machine (SVM), used for categorization and regressive analysis, is a monitored machine learning technology extensively. And we used RFE arithmetic to screen the optimal genes from the metadata. We validated the expression of key candidate diagnostic markers with GSE169077.

### Immune infiltration analysis

Using CIBERSORT (http://cibersort.stanford.edu/), we identified the immune response of 22 kinds of immune cells and evaluated the association between the expression of key genes and these immune cells in normal and osteoarthritic samples.

### Receiver operating characteristic (ROC) analysis of the diagnostic effect

After we obtained GSE29746 from the GEO database, the R glm function was used to build the logistics model, the pROC package was used for analysis, and the ggplot2 package was used for visualization to plot the ROC.

### Animal models of OA

There were 20 C57BL/6J mice weighing 20 ± 2 g, and they were randomly divided into a normal group and an OA group. In the OA group, we successfully constructed an OA model by referring to Hulth’s method: the medial collateral ligament, anterior and posterior cruciate ligament, and medial meniscus were removed. Postoperative animals were routinely kept in cages. One week after surgery, rats were given 2 doses daily and forced to be active for 30 min, and OA models could be established after 4 weeks. Then, all animals were killed using CO_2_ euthanization methods. Then, we collected knee tissues, fixed them in 4% paraformaldehyde for 48 h and decalcified them in EDTA decalcification solution for 1 month. The cartilage tissues of each group were embedded and sliced (5 μm) in the sagittal plane. The prepared paraffin sections were deparaffinized with xylene and then passed through 100%, 100%, 95%, 90%, 80%, and 70% alcohol. Then, the slices were stained with haematoxylin and eosin (Beijing Solarbio Science and Technology Co., Ltd.) in sequence, and finally, the slices were sealed with neutral gum.

### qRT‒PCR

LncRNA and mRNA primers were designed and synthesized by Shanghai Shenggong Biological Co., Ltd. (β-actin: forward (GGCAGCGGCAGGATACAC) and reverse (TTCACAGGACACGAGCTG); Lama2: forward (TCCCAAGCGCATCAACAGAG) and reverse (CAGTACATCTCGGGTCCTTTTTC); Nid2: forward (ACTGCCAGTCGAGGTTTTACG) and reverse (GACCACTCACTTTCCCATTCAC); Xist: forward (CATCCGCTTGCGTTCATAGT) and reverse (ACCGCTTGAGATCAGTGCTG); TTTY15: forward (TCTATGACCTGGAAGC) and reverse (ATCTGATGGAACCCTA)) and β-actin was used as an internal reference control. MiRNA quantification: Bulge-loop™ miRNA qRT‒PCR Primer Sets (one RT primer and a pair of qRT‒PCR primers for each set) specific for miR-29a-3p, miR-29b-3p and let-7a-5p were designed by RiboBio (Guangzhou, China). Total RNA from the NPCs was extracted using kit reagents (Vazyme, China, RC112-01) according to the manufacturer’s instructions. Reverse transcription of 1 µg of RNA enables the amplification of complementary products. Quantitative PCR was performed in a 20 µL reaction system containing specific primers and ChamQ SYBR qPCR Master Mix (Vazyme, China, Q321-02). Amplification was performed in the Roche LightCycler 480 System (Roche, Switzerland). The PCR extension conditions were 95 °C for 30 s, 95 °C for 5 s, 60 °C for 34 s, and 40 cycles. There were three replicates for each group. The Ct value obtained for each group is represented by 2-ΔΔCt.

### LncRNA‒miRNA‒mRNA network prediction

Six databases, TargetScan, miRTarBase, miRDB, RNAInter, TargetMiner and RNA22, were used to predict the target miRNAs of LAMA2 and NID2, and we obtained the final miRNA of interest by taking the intersecting data. We used the ENCORI database (https://starbase.sysu.edu.cn/index.php) [17] to predict the targeting lncRNAs of miRNAs. Then, the DIANA database (https://diana.e-ce.uth.gr/home) was used to verify the interaction between miRNAs and lncRNAs. Finally, we used Cytoscape (version 3.9.1) for visualization.

### Statistical analysis

Student’s t test was used to compare gene expression in osteoarthritic specimens with that in healthy specimens. There was a statistically significant difference when ∗∗∗∗p < 0.0001, ∗∗∗p < 0.001, ∗∗p < 0.01, or ∗p < 0.05.

## Results

### Identification of DEGs

**Fig. 1:**
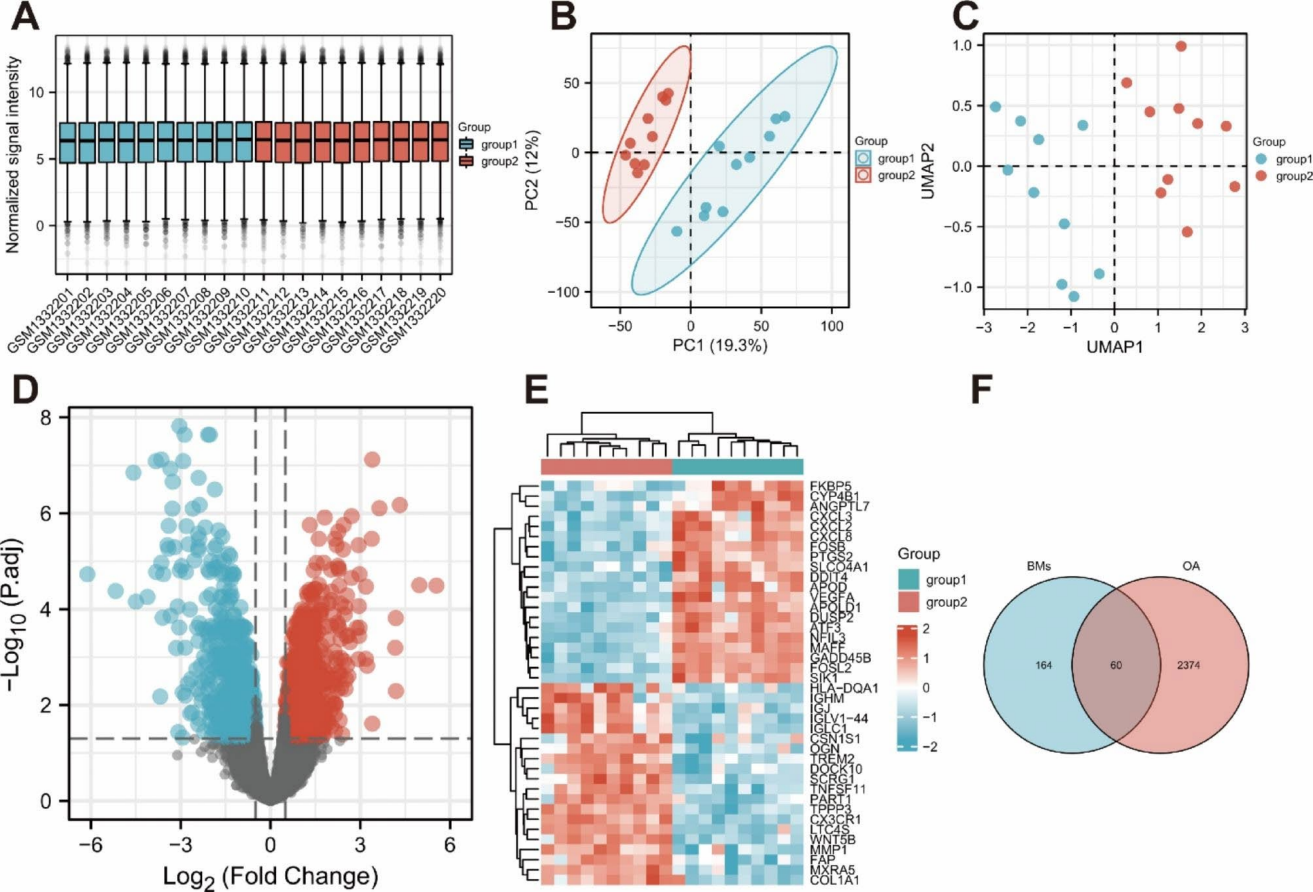
Identification of DEGs. **A**: Box plot after normalization of GSE55235; **B**: PCA graph; **C**: UMAP graph; **D**: Volcano plot; **E**: Heatmap of expression of the top 20 genes with high and low expression; **F**: Venn diagram

The GSE55235 information was obtained from the GEO Database. Through R language, the corresponding data were downloaded from GEO, and difference analysis was performed. Through the box plot (Fig. 1A), we found that the median of each sample was basically on a horizontal line, indicating that the degree of normalization between samples is good. From the PCA graph (Fig. 1B) and the UMAP graph (Fig. 1C), there were significant differences between the two groups. Afterwards, a p value < 0.05 and a |log FC| value > 0.5 were used as the criteria for identifying DEGs, and a volcano plot was generated (Fig. 1D). A total of 1270 upregulated DEGs and 1164 downregulated DEGs were obtained. Finally, a heatmap was made (Fig. 1E) to confirm that the expression of the top 20 genes with high and low expression in the expression profile showed significant differences between the two groups.

We obtained BM-related genes from previous research reports [11]. By taking the intersection with the above DEGs, we obtained 60 BM-related DEGs (Fig. 1F).

### KEGG/GO enrichment analysis

**Fig. 2:**
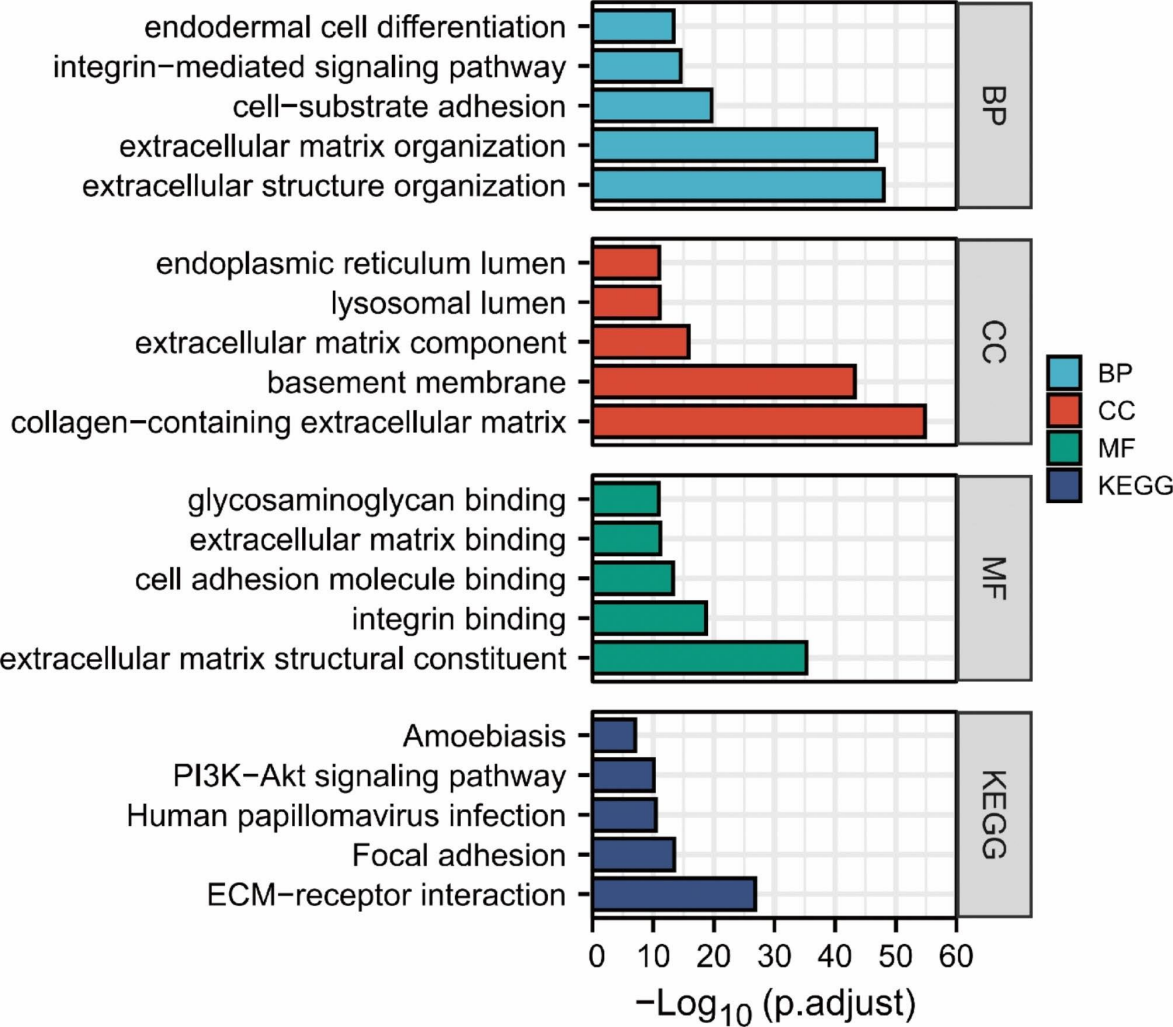
Top 5 results of GO/KEGG analysis of DEGs

Under the conditions of p.adj < 0.05 and qvalue < 0.2, the enrichment results showed that there were a total of 310 BPs, which were mainly involved in extracellular structure and matrix organization. The CC category mainly showed enrichment in the extracellular matrix, basement membrane, lysosomal lumen, and endoplasmic reticulum lumen. MF mainly showed enrichment in extracellular matrix structural constituent, integrin binding, cell adhesion molecule binding, extracellular matrix binding, and glycosaminoglycan binding. KEGG pathway enrichment showed a total of 23 pathways, mainly ECM-receptor interaction, focal adhesion, and the PI3K-Akt signalling pathway (Fig. 2).

### Identification of diagnostic marker candidates for OA

**Fig. 3:**
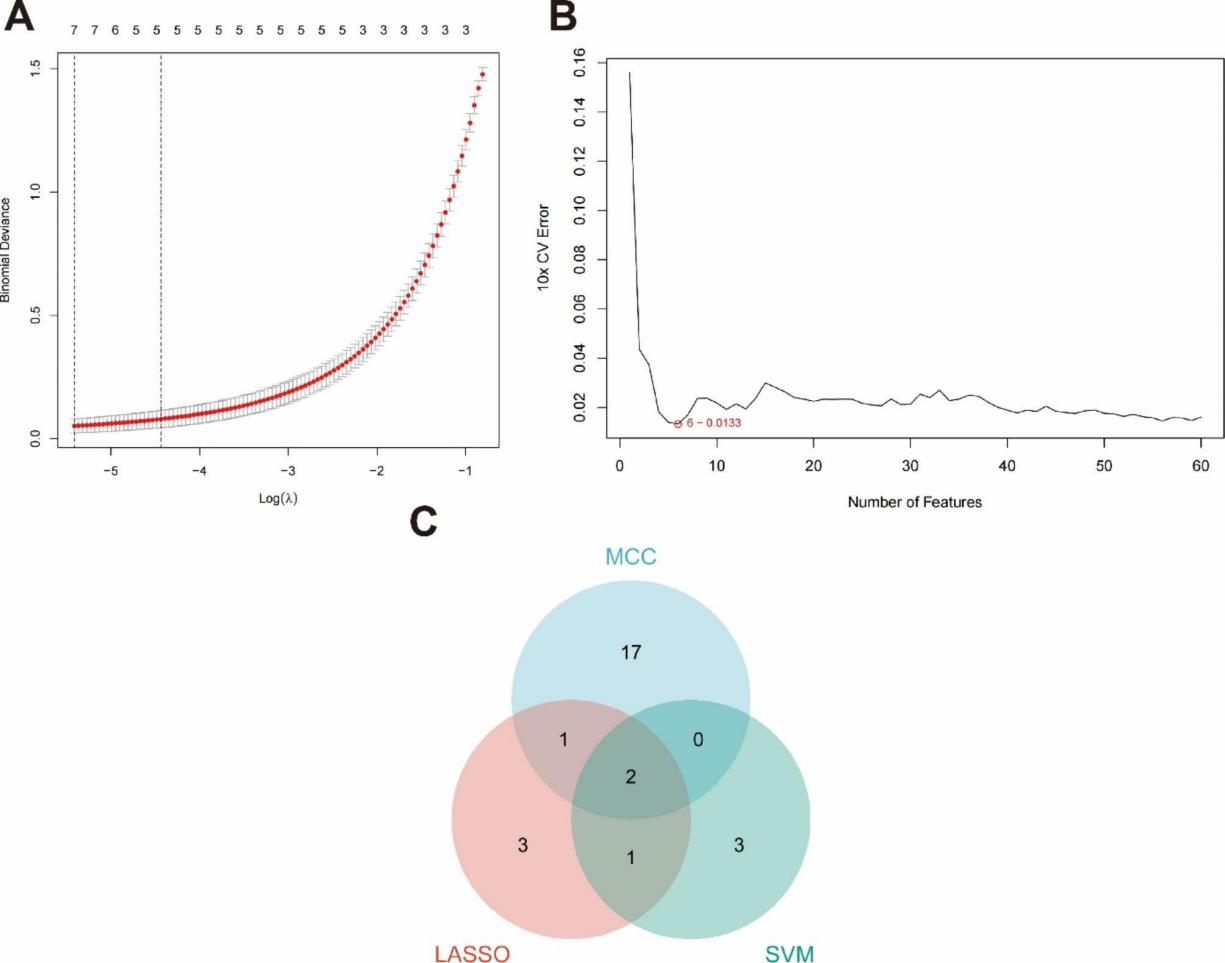
Selection of diagnostic marker candidates for OA. **A**: Tuning feature screening in the LASSO model; **B**: A plot of biological marker screening via the SVM-RFE arithmetic; **C**: Venn graph displaying 2 diagnosis biomarkers shared by LASSO and SVM-RFE

We used the MCC algorithm of the cytoHubba plugin in Cytoscape software to obtain the top 20 scoring genes (Table 1). Then, 7 diagnostic genes were obtained by the LASSO regression algorithm (Fig. 3A), and 6 diagnostic genes were obtained by SVM-RFE (Fig. 3B). We obtained two crossover genes: LAMA2 and NID2. LAMA2 was downregulated in osteoarthritic tissue, and NID2 was upregulated in osteoarthritic tissue (Fig. 3C).

**Table 1:** The top 20 genes scored by MCC algorithm

Gene	Expression	Gene	Expression	Gene	Expression	Gene	Expression
LAMA5	DOWN	LAMB1	DOWN	COL5A1	UP	THBS1	DOWN
COL6A1	UP	LAMA4	UP	NID1	DOWN	NID2	UP
ITGA5	DOWN	LAMA3	DOWN	ITGA4	UP	LAMB4	DOWN
ITGAV	UP	HSPG2	UP	ITGB5	UP	SPARC	UP
LAMA2	DOWN	ITGA8	DOWN	FBN1	UP	BGN	UP

### Immune cell infiltration analysis

**Fig. 4:**
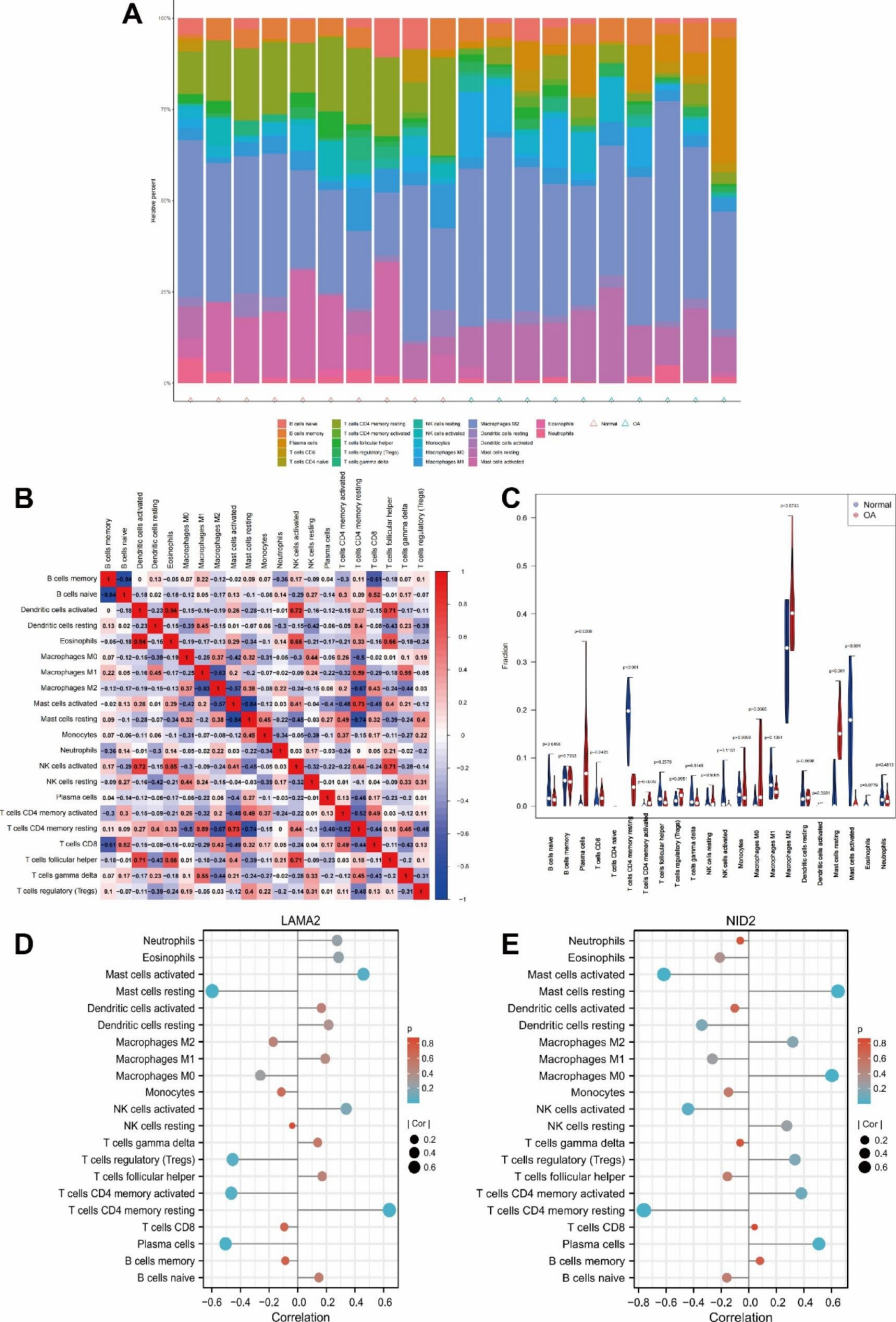
Immune cell infiltration analysis of OA and hub genes. **A-B**: The percentage of the immunocytes identified via the CIBERSORT algorithm. **C**: The immunocytes between healthy and OA specimens. **D-E**: Correlation between LAMA2, NID2 and immune cells in OA and normal samples

An increasing number of studies have shown the role of immune infiltration in OA. Our team used the CIBERSORT algorithm to study the difference in immune cells between the OA group and the normal group and the relationship between LAMA2 and NID2 and immune cells. The relationship between the immune cells of the OA group and the normal group is shown in Fig. 4A and B. In addition, plasma cells, CD8 T cells, resting memory CD4 T cells, activated memory CD4 T cells, resting mast cells, and activated mast cells were significantly different in expression between the OA and normal sample groups (Fig. 4C). Furthermore, we investigated the relationship between LAMA2 and NID2 and the levels of immune infiltration. LAMA2 was associated with resting memory CD4 T cells, resting mast cells, plasma cells, activated memory CD4 T cells, activated mast cells, and regulatory T cells (Fig. 4D). NID2 was closely related to resting memory CD4 T cells, resting mast cells, activated mast cells, M0 macrophages, and plasma cells (Fig. 4E). Our study suggested that LAMA2 and NID2 may be involved in OA initiation and progression by regulating some immune cells.

### Gene expression validation

**Fig. 5:**
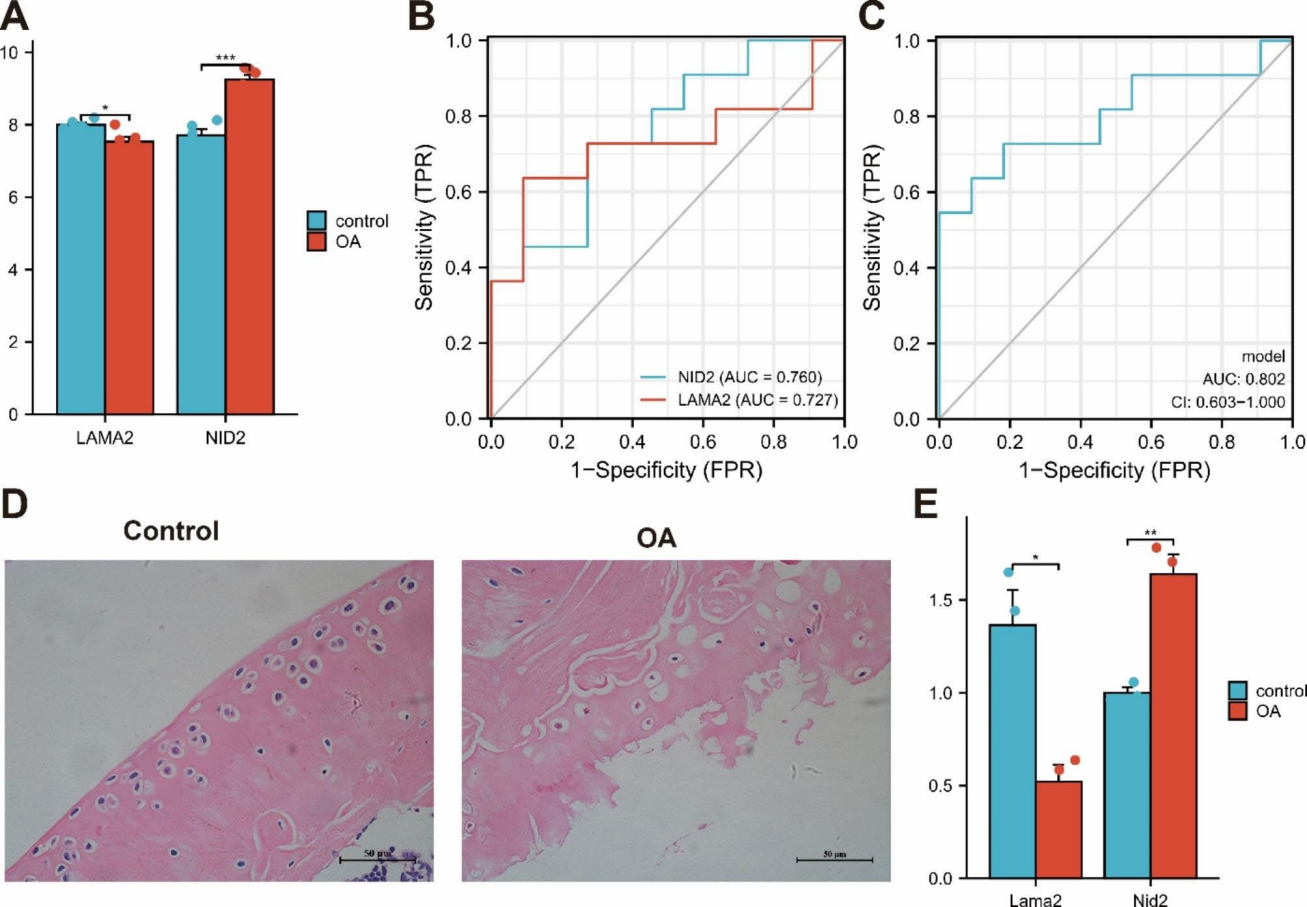
Diagnostic value and expression validation of diagnostic markers. **A**: The expression of LAMA2 and NID2 in GSE169077. **B**: ROC of LAMA2 and NID2 in GSE29746. **C**: ROC of LAMA2 + NID2 in GSE29746. **D**: H&E staining of models. E: The expression of LAMA2 and NID2 (∗∗∗p < 0.001, ∗∗p < 0.01, or ∗p < 0.05)

GSE169077 was used to examine the expression of LAMA2 and NID2, and the results showed that LAMA2 was expressed at lower levels and NID2 was expressed at higher levels in OA, which was consistent with the results of the above analysis (Fig. 5A). GSE29746 data included 11 healthy tissue samples and 11 OA tissue samples. Our studies show that NID2 (Fig. 5B, AUC = 0.760) and LAMA2 (Fig. 5B, AUC = 0.727) have good diagnostic effects as separate diagnostic indicators. In addition, we constructed a joint index model, and the results showed that LAMA2 and NID2 as joint indicators had better diagnostic effects than a single gene (Fig. 5C, AUC = 0.802). Additionally, in our OA model, by H&E staining, we observed that the cartilage surface was rough and thinned and that the lamellar structure disappeared. The number of chondrocytes decreased, the size was different, and the arrangement was disordered (Fig. 5D), which showed that mould building was successful. Furthermore, lower expression of LAMA2 and higher expression of NID2, verified by qRT‒PCR, were detected compared to healthy tissue samples (Fig. 5E).

### LncRNA‒miRNA‒mRNA network prediction

**Fig. 6:**
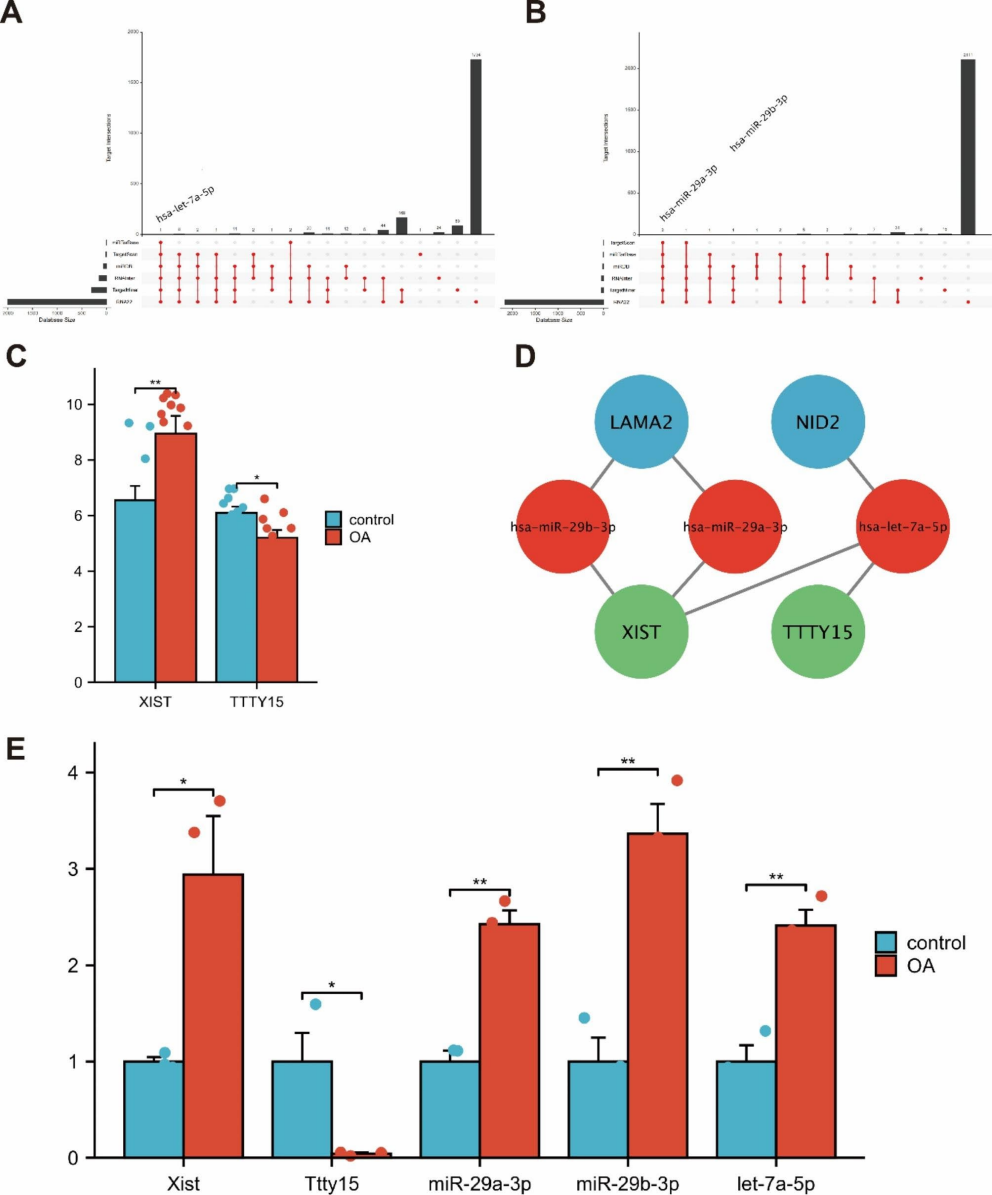
LncRNA‒miRNA‒mRNA network prediction and miRNA and lncRNA expression verification. **A**: The miRNAs interacting with NID2. **B**: The miRNAs interacting with LAMA2. **C**: The expression of XIST and TTTY15 (∗p < 0.05，∗∗p <0.01). **D**: LncRNA-miRNA-mRNA network. **E**: The expression of XIST, TTTY15, miR-29a-3p, miR-29b-3p and let-7a-5p (∗∗p < 0.01, or ∗p < 0.05)

The miRNAs that interacted with mRNA were predicted from six databases, and they were hsa-miR-29a-3p, hsa-miR-29b-3p and hsa-let-7a-5p (Fig. 6A-B). Moreover, the relevant lncRNAs XIST and TTTY15 were predicted by the ENCORI database and the DIANA database. The expression of these two genes in the GSE55235 dataset was significantly different between OA samples and healthy samples (Fig. 6C). We also obtained their interaction relationship (Fig. 6D). We also verified the expression of these genes by qRT‒PCR. The expression of XIST and TTTY15 was consistent with the results of dataset analysis; XIST was highly expressed in the OA group, and TTTY15 was expressed at low levels in the OA group. The results also showed that all three miRNAs were highly expressed in the OA group (Fig. 6E).

## Discussion

Osteoarthritis is considered the most prevalent chronic joint disease and is a major source of disability, pain, and socioeconomic costs worldwide. However, the diagnosis of osteoarthritis is often made late in the disease process, and it is too late for effective treatment [18, 19]. In this paper, bioinformatics was used to study the differential expression of genes in OA samples and healthy samples, and we conducted GO/KEGG enrichment analysis of differentially expressed genes. These genes were indeed mainly concentrated in the extracellular matrix and basement membrane, and the signalling pathway was also mainly concentrated in extracellular matrix receptor interactions. We also used PPI network analysis, LASSO regressive arithmetic and the SVM-RFE algorithm to obtain two high-value BM-related genes as candidate diagnostic markers: LAMA2 and NID2, which are important biomarkers highly correlated with the occurrence and development of OA. Then, we used the GSE169077 dataset to verify the expression of these two genes, and the conclusions obtained showed that was consistent with the results of the above analysis. GSE29746 dataset was used to verify the diagnostic value of them, and the conclusions obtained showed that the diagnostic effect of the two genes as a combined indicator was better than that of the single gene. Moreover, the expression of LAMA2 and NID2 has been verified in animal models. Furthermore, the CIBERSORT algorithm was used to calculate the immune infiltration between the two groups of samples and the relationship between two BM-related hub genes and immune cells. Finally, we predicted the lncRNA‒miRNA‒mRNA network associated with these potentially important genes, and we validated their expression in OA.

BMs are an extracellular matrix with a reticular structure located beneath epithelial cells and are formed by two main macromolecule proteins, laminin and collagen type IV [20]. BMs play an important role in many developmental processes, such as polarity, signalling, differentiation, tissue maintenance and shaping and connecting tissues [21]. Moreover, defects in the composition or assembly of BMs can lead to various diseases, such as cancer and fibrosis [22, 23].

We used a variety of machine learning methods to obtain hub genes associated with BMs: LAMA2 and NID2. Among them, NID2 has been confirmed by a previously published article, which shows that NID2 was increased in osteoarthritic cartilage, and NID2 could promote chondrogenesis by influencing the antagonism between SOX9 and RUNX2, increasing SOX9 and promoting the synthesis of type II collagen [24]. The laminin alpha 2 chain is encoded by the LAMA2 gene, which is also related to hip dysplasia and cartilage development and is thought to contribute to congenital muscular dystrophy [25, 26]. Our research also showed that LAMA2 expression was downregulated in OA. Our study also showed that LAMA2 and NID2, as joint indicators, can significantly improve the diagnostic effect of OA. These two hub genes can be used as important potential targets for the diagnosis and treatment of OA.

We conducted immune infiltration analysis and found that CD4 memory resting T cells, resting mast cells, activated mast cells, and plasma cells were dysregulated in OA and healthy samples. Interestingly, both LAMA2 and NID2 were also closely related to these immune cells. In OA samples, resting memory CD4 T cells were downregulated, and activated memory CD4 T cells were upregulated. Moreover, LAMA2 was positively correlated with resting CD4 memory T cells and negatively correlated with activated CD4 memory T cells, and NID2 was the opposite, while LAMA2 was poorly expressed and NID2 was highly expressed in OA samples. These results suggested that LAMA2 and NID2 may promote the development of OA by participating in the regulation of memory CD4 T cells. At present, studies have also shown that the joints of OA patients have CD4 + T-cell infiltration, which increases the secretion of immunomodulatory cytokines, aggravates local inflammation, and aggravates the OA process [27, 28]. F Ponchel et al. also showed that memory CD4 + T cells increased in blood samples of OA patients compared with healthy subjects [27], which was consistent with our analysis results. Mast cells were also shown to be associated with structural damage in patients with OA in B J E de Lange-Borkar’s research [29]. The role of these immune cells in OA remains to be further studied and is expected to become a potential mechanism and potential therapeutic target for the development of OA. In addition, we conducted lncRNA‒miRNA‒mRNA network prediction. XIST was significantly decreased in OA, and TTTY15 was significantly upregulated in OA compared to healthy samples. Moreover, the analysis showed the existence of the XIST-hsa-miR-29a-3p/hsa-miR-29b-3p-LAMA2 and XIST/TTTY15-hsa-let-7a-5p-NID2 network relationships. This finding provides more possibilities for the diagnosis and treatment of OA.

Of course, our study also has some limitations, such as the specific regulatory mechanisms of immune cells and genes and the relationship between lncRNA‒miRNA‒mRNA regulation and the occurrence and progression of OA, which need to be further validated in human tissues.

## Conclusion

Through biological information analysis and machine learning, we obtained two crucial BM-related genes, LAMA2 and NID2, and they were tested with animal experiments. Moreover, immune infiltration analysis was performed, indicating that memory CD4 T cells, resting mast cells, activated mast cells, and plasma cells showed significant differences between the OA samples and the healthy samples and were highly correlated with BM-related genes. Finally, we identified two different lncRNAs in OA, XIST and TTTY15, and predicted their relationship with the lncRNA‒miRNA‒mRNA network of hub genes. The expression of these lncRNAs and miRNAs was also verified. These hub genes, immune cells and related network relationships indicated that BMs play an important role in the occurrence and development of OA, and they can be used for new research on the pathogenesis of OA and potential therapeutic targets.

## Data Availability

The datasets generated and analysed during the current study are available in the GEO repository(https://www.ncbi.nlm.nih.gov/geo/). GSE55235 (https://www.ncbi.nlm.nih.gov/geo/query/acc.cgi?acc=GSE55235), GSE169077 (https://www.ncbi.nlm.nih.gov/geo/query/acc.cgi?acc=GSE169077), GSE29746 (https://www.ncbi.nlm.nih.gov/geo/query/acc.cgi?acc=GSE29746).
